# Gastric cancer risk after *Helicobacter pylori* eradication in gastritis and peptic ulcer: a retrospective cohort study in Japan

**DOI:** 10.1186/s12876-025-04034-3

**Published:** 2025-07-01

**Authors:** Kentaro Sugano, Chihiro Suzuki, Mihoko Ota, Ryuichi Iwakiri

**Affiliations:** 1 Jichi Medical University, Tochigi, Japan; 2 Japan Medical Office, Takeda Pharmaceutical Company Limited, 1-1, Nihonbashi-Honcho 2-Chome, Chuo-Ku, Tokyo, 103-8668 Japan; 3 Department of Gastroenterology, Shin Komonji Hospital, Fukuoka, Japan; 4 Present Address: Institute of Science Tokyo, Tokyo, Japan

**Keywords:** Claims database, Duodenal ulcer, Eradication therapy, Gastric atrophy, Gastric cancer, Gastric ulcer, Gastritis, Helicobacter pylori

## Abstract

**Background:**

*Helicobacter pylori* infection is an important risk factor for gastric cancer. In Japan, national health insurance has covered eradication therapy for *H. pylori* infection–associated gastritis from 2013. However, gastric cancer was the fourth leading cause of cancer death in 2023. We aimed to investigate differences in gastric cancer risk among patients with gastritis, gastric ulcer, duodenal ulcer, and gastric ulcer and duodenal ulcer after *H. pylori* eradication.

**Methods:**

This retrospective cohort study used the JMDC Claims Database from February 21, 2013, to August 31, 2023. Patients who received first-line *H. pylori* eradication therapy and were diagnosed with *H. pylori*–associated gastritis, gastric ulcer, or duodenal ulcer in the same month or the month before the first eradication therapy prescription were included. Two antibacterial drugs and an acid secretion inhibitor or triple-drug blister-packaged product were prescribed. The primary outcome was gastric cancer incidence. A Cox proportional hazards regression analysis was used to estimate hazard ratios (HRs). A propensity score approach was used to minimize the effect of confounding measures.

**Results:**

Of 17,245,330 beneficiaries, 148,489 were included. In the weighted cohort (after propensity matching), statistically significant differences were observed in HRs between *H. pylori*–associated gastritis and duodenal ulcer (HR using the latter as a reference [95% confidence interval]: 2.03 [1.31–3.13]; *p* = 0.001), and between gastric ulcer and duodenal ulcer (2.37 [1.52–3.71]; *p* < 0.001). The cumulative probabilities (95% confidence interval) per the median follow-up years (3.8 years for all) were 0.44% (0.39–0.48) for *H. pylori*–associated gastritis, 0.54% (0.46–0.63) for gastric ulcer, 0.22% (0.10–0.33) for duodenal ulcer, and 0.26% (0.08–0.50) for gastric ulcer and duodenal ulcer.

**Conclusions:**

Patients with *H. pylori*–associated gastritis and gastric ulcer had a higher risk of gastric cancer than patients with duodenal ulcer, indicating that gastric atrophy remains a risk factor after *H. pylori* eradication therapy. Careful monitoring, such as by endoscopic examination, is required after successful eradication of *H. pylori* in patients at higher risk.

**Supplementary Information:**

The online version contains supplementary material available at 10.1186/s12876-025-04034-3.

## Introduction

*Helicobacter pylori* infection is a risk factor for gastric ulcer (GU), duodenal ulcer (DU), gastric mucosa–associated lymphoid tissue (MALT) lymphoma, and idiopathic thrombocytopenic purpura following gastritis [1]. Furthermore, *H. pylori* infection is considered to be an important risk factor for gastric cancer [2, 3]. In Japan, national health insurance has covered *H. pylori* eradication therapy for *H. pylori*–associated gastritis (HPAG) from 2013 [1], in addition to GU and DU. However, gastric cancer was the fourth most common cause of cancer death in Japan in 2023 following lung cancer, colon/rectum cancer, and pancreatic cancer among all cancer patients [4].

*H. pylori* infection is involved in chronic inflammation of the gastric mucosa, which is thought to progress to gastric atrophy and intestinal metaplasia of the gastric mucosa and can develop into gastric cancer over time [1, 5]. The degree of gastric atrophy and the distribution of *H. pylori* in conjunction with histological gastritis vary among HPAG, GU, and DU [6–8]. Because *H. pylori* eradication is strongly indicated for these diseases [1], it is important to evaluate the incidence of gastric cancer after *H. pylori* eradication. Previous studies have reported that patients with severe atrophic gastritis at baseline have an increased risk of gastric cancer after *H. pylori* eradication [9–11] because the gastric atrophy did not improve in the short term. A large prospective study post *H. pylori* eradication in patients with peptic ulcer showed that GU but not DU was a risk factor for gastric cancer [12]. However, evaluation of the difference in gastric cancer risk among patients with HPAG, GU, DU, and gastric ulcer and duodenal ulcer (GDU) after *H. pylori* eradication is limited [9, 13].

This retrospective cohort study using data from the JMDC Claims Database aimed to investigate differences in the gastric cancer risk among patients with HPAG, GU, DU, and GDU after *H. pylori* eradication.

## Materials and methods

### Study design

**Fig. 1:**
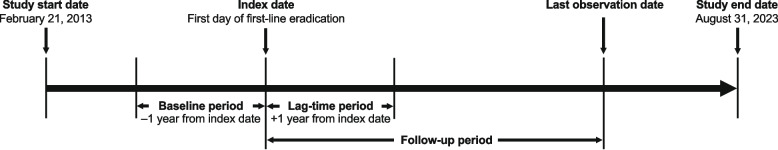
Study design

This was a retrospective cohort study using claims data from the JMDC Claims Database from February 21, 2013, to August 31, 2023 (Fig. 1). In Japan, the national health insurance has covered *H. pylori* eradication therapy for *H. pylori* infection–associated gastritis from February 21, 2013 [1], in addition to providing insurance coverage for patients with GDU; therefore, data from February 21, 2013, were used for the study.

The JMDC Claims Database is an epidemiological claims database that includes claims of inpatient, outpatient, dispensing, and health checkup data received from multiple health insurance associations in Japan since 2005. Data are cumulated from approximately 17 million beneficiaries (as of August 2024), including health insurance associations of major companies and people living in large cities.

Data based on the International Classification of Diseases 10th Revision (ICD-10) and receipt code–defined target disease were extracted from the database for the study. For patients diagnosed with HPAG (ICD-10: K29.3 [chronic superficial gastritis], K29.4 [chronic atrophic gastritis], K29.5 [chronic gastritis, unspecified]; receipt codes: 8841341 [*H. pylori* gastritis], 8845818 [intestinal metaplasia]), GU (ICD-10: K25.X), or DU (ICD-10: K26.X), the index date was the date of the first prescription for first-line *H. pylori* eradication therapy. The index month was defined as the month of the index date. The baseline was defined as a period from the day 1 year before the index date to the index date. If multiple data were present during baseline, the data that were closest to the index date were used. The lag-time period was up to 1 year after the index date. Follow-up period was from the index month to the last observation month. Gastric cancer was defined as a disease diagnosed as C16.X based on the ICD-10 code.

This was a retrospective study using data from a claims database. Because of the anonymous nature of the analysis and the absence of direct patient involvement, ethical approval and informed patient consent were not required, based on the Ethical Guidelines for Epidemiological Research issued by the Japanese Ministry of Health, Labour and Welfare [14]. The study conformed to the ethical guidelines of the Declaration of Helsinki.

### Study population

The study population consisted of patients who met all of the following inclusion criteria: received first-line *H. pylori* eradication therapy consisting of two antibacterial drugs (amoxicillin + clarithromycin) and an acid secretion inhibitor (proton pump inhibitor or potassium-competitive acid blocker) that were prescribed on the same day for 7 days or a triple-drug blister-packaged product that was prescribed for 7 days; diagnosed with HPAG, GU, or DU in the same month or the month before the first day of first-line eradication therapy prescription; and *H. pylori* test, either non-invasive (e.g., urea breath test, anti–*H. pylori* antibody test, and stool *H. pylori* antigen test) or invasive requiring an endoscopy (e.g., nucleic acid enhancement test, rapid urease test, histology, and culture test), performed on the first day of first-line eradication treatment prescription or within 3 months before the first prescription. Patients who met one of the following criteria were excluded from the study population: aged < 18 years as of the index month; diagnosed with idiopathic thrombocytopenic purpura or gastric MALT lymphoma within 1 year before index month; unable to track patient data 1 year before or after index month; diagnosed with cancer within 1 year before or after index month; had an endoscopic gastric or duodenal polyp/mucosal resection to remove malignant tumors within 1 year before index month; or had a gastrectomy within 1 year before index month.

### Outcomes

The primary endpoint of this study was the incidence of gastric cancer. The secondary endpoint of this study was the cumulative probability of development of gastric cancer at median follow-up period. Patient demographics—including sex, age, body mass index (BMI), smoking history, low-dose aspirin, endoscopies, second-line eradication, and index year—were summarized.

### Statistical analysis

#### Sample size

The study was conducted using the JMDC Claims Database, and therefore sample size was not set and all data in the database that met the predefined criteria were included in the analysis. At the time of drafting the study protocol, based on the ICD-10 codes for the defined target diseases, the number of confirmed patients within the database was 3,621,944.

#### Data analysis

Analysis results are provided by HPAG, GU, DU, and GDU. If both HPAG and GU apply, the patients were categorized as GU patients, and if both HPAG and DU apply, the patients were categorized as DU patients.

For continuous variables, summary statistics were calculated, together with the total number of observations and the number of missing values. For categorical variables, the number of patients and their proportion were calculated for each category. Standardized differences between disease groups were calculated for baseline characteristics. A propensity score approach was used to minimize the effect of measured confounding on the results. A multinomial logistic model was used to calculate the probability of being diagnosed with HPAG, GU, DU, and GDU to address the initial confounding among the four diseases. Propensity scores were estimated using a multinomial logistic model with the four diseases as dependent variables and the covariates (sex, age, BMI, smoking history, low-dose aspirin, and index year) as independent variables. Analysis was performed using inverse probability weighting. Stabilized weight was used to reduce the impact of extreme weights and variance of estimated effects. A multiple imputation by chained equation was used to impute missing values for variables included in the propensity score model. All analyses were conducted for both the crude and weighted populations. The two-sided significance level of the test was 5%, and confidence intervals (CIs) were calculated at two-sided 95%. For the primary outcome, the hazard ratios (HRs) and their 95%CIs among HPAG, GU, DU, and GDU were estimated by Cox proportional hazards regression analysis using robust variance, with the diseases as the only covariates. For the secondary outcome, the cumulative probability curve of development of gastric cancer was drawn using the Kaplan–Meier method, and the cumulative probability and their 95%CIs at the median follow-up period were calculated. Event was defined as diagnosis with gastric cancer (ICD-10: C16.X) after lag time. Data were censored at the end of the month of the follow-up period or the end of the month of study, whichever was earlier, after lag time. Sensitivity analyses were conducted with the lag time set to 2 and 3 years. Multiple testing adjustments were not made. SAS® 9.4 was used for statistical analysis (SAS Institute Inc., Cary, NC, USA).

## Results

### Study population

**Fig. 2:**
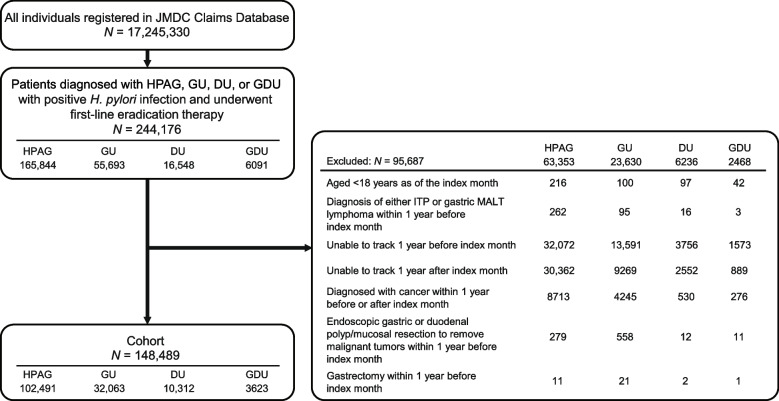
Patient flowchart of the crude cohort. DU, duodenal ulcer; GDU, gastric ulcer and duodenal ulcer; GU, gastric ulcer; HPAG, *Helicobacter pylori*–associated gastritis; ITP, idiopathic thrombocytopenic purpura; MALT, mucosa-associated lymphoid tissue

The number of beneficiaries who were registered in the JMDC Claims Database from February 2013 to August 2023 was 17,245,330 (Fig. 2). Of those, 244,176 patients met the inclusion criteria and 148,489 were included in the crude cohort, which consisted of patients with HPAG (*n* = 102,491), GU (*n* = 32,063), DU (*n* = 10,312), and GDU (*n* = 3623); 95,687 patients were excluded from the analysis. The most common reasons for exclusion were “not tracking 1 year before or after the index month” and “diagnosis of cancer within 1 year before or after the index month” in each group.

### Baseline demographics and characteristics

The proportion of male patients was > 50% in all groups, with the highest proportions in the GDU group (Table 1). The mean age was > 46 years, with the highest age in the GU group. The proportion of habitual smokers was lowest in the HPAG group (22.04%) and highest in the GDU group (44.32%). Distribution of BMI, proportion of patients prescribed low-dose aspirin, and proportion of patients who underwent second-line *H. pylori* eradication were similar between groups. After matching, all standardized differences in key baseline characteristics were < 0.1, indicating that the disease groups were well balanced after adjustment (Table A1).

**Table 1:** Baseline demographics and characteristics

	**HPAG**	**GU**	**DU**	**GDU**
	***N* (%)**	***N* (%)**	***N* (%)**	***N* (%)**
**Crude cohort**				
Total	102,491	32,063	10,312	3623
Sex				
Male	60,025 (58.57)	20,268 (63.21)	7555 (73.26)	2789 (76.98)
Female	42,466 (41.43)	11,795 (36.79)	2757 (26.74)	834 (23.02)
Age, years				
Mean (SD)	49.0 (10.2)	49.8 (10.5)	46.2 (10.7)	47.5 (10.9)
Median	49.0	50.0	47.0	48.0
Q1–Q3	41.0–56.0	43.0–57.0	39.0–54.0	40.0–55.0
Min–max	18–74	18–74	18–73	18–74
< 50	52,051 (50.79)	15,027 (46.87)	6268 (60.78)	1971 (54.40)
50–59	33,672 (32.85)	11,130 (34.71)	2944 (28.55)	1170 (32.29)
≥ 60	16,768 (16.36)	5906 (18.42)	1100 (10.67)	482 (13.30)
BMI, kg/m^2a^				
< 18.5	5358 (6.32)	1855 (7.31)	446 (5.54)	158 (5.68)
18.5– < 25.0	56,007 (66.04)	16,292 (64.16)	5459 (67.81)	1768 (63.60)
25.0– < 30.0	19,195 (22.63)	5913 (23.29)	1795 (22.30)	696 (25.04)
≥ 30.0	4244 (5.00)	1331 (5.24)	350 (4.35)	158 (5.68)
Unknown	17,687 (NA)	6672 (NA)	2262 (NA)	843 (NA)
Smoking^a^				
Habitual smoking	18,175 (22.04)	8651 (34.98)	3020 (38.86)	1193 (44.32)
Non-habitual smoking	64,299 (77.96)	16,083 (65.02)	4751 (61.14)	1499 (55.68)
Unknown	20,017 (NA)	7329 (NA)	2541 (NA)	931 (NA)
Low-dose aspirin				
Presence	93 (0.09)	63 (0.20)	8 (0.08)	6 (0.17)
Absence	102,398 (99.91)	32,000 (99.80)	10,304 (99.92)	3617 (99.83)
Endoscopies				
Presence	10,031 (9.79)	5643 (17.60)	1786 (17.32)	808 (22.30)
Absence	92,460 (90.21)	26,420 (82.40)	8526 (82.68)	2815 (77.70)
Second-line eradication				
Presence	9153 (8.93)	2896 (9.03)	750 (7.27)	283 (7.81)
Absence	93,338 (91.07)	29,167 (90.97)	9562 (92.73)	3340 (92.19)
Index year				
2013	0 (0.0)	0 (0.0)	0 (0.0)	0 (0.0)
2014	5318 (5.19)	2777 (8.66)	793 (7.69)	328 (9.05)
2015	6235 (6.08)	2770 (8.64)	878 (8.51)	321 (8.86)
2016	9225 (9.00)	3373 (10.52)	1070 (10.38)	423 (11.68)
2017	11,420 (11.14)	3877 (12.09)	1217 (11.80)	445 (12.28)
2018	14,487 (14.13)	4412 (13.76)	1439 (13.95)	507 (13.99)
2019	16,255 (15.86)	4575 (14.27)	1523 (14.77)	535 (14.77)
2020	14,002 (13.66)	3764 (11.74)	1277 (12.38)	411 (11.34)
2021	15,897 (15.51)	4087 (12.75)	1323 (12.83)	399 (11.01)
2022	9652 (9.42)	2428 (7.57)	792 (7.68)	254 (7.01)
**Weighted cohort**				
Total	102,282	31,867	10,336	3570
Sex				
Male	62,397 (61.01)	19,435 (60.99)	6183 (59.82)	2167 (60.70)
Female	39,885 (38.99)	12,432 (39.01)	4153 (40.18)	1403 (39.30)
Age, years				
Mean (SD)	49.0 (10.2)	48.9 (10.6)	49.5 (10.4)	49.6 (10.5)
Median	49.0	49.0	50.0	50.0
Q1–Q3	41.0–56.0	42.0–57.0	42.0–57.0	43.0–57.0
Min–max	18–74	18–74	18–73	18–74
< 50	52,073 (50.91)	15,988 (50.17)	5007 (48.45)	1659 (46.45)
50–59	33,600 (32.85)	10,548 (33.10)	3517 (34.03)	1282 (35.89)
≥ 60	16,609 (16.24)	5331 (16.73)	1811 (17.52)	630 (17.65)
BMI, kg/m^2a^				
< 18.5	5264 (6.20)	1837 (7.27)	465 (5.65)	179 (6.48)
18.5– < 25.0	56,150 (66.15)	16,158 (63.94)	5473 (66.54)	1765 (64.06)
25.0– < 30.0	19,287 (22.72)	5880 (23.27)	1886 (22.93)	660 (23.96)
≥ 30.0	4181 (4.93)	1393 (5.51)	401 (4.87)	152 (5.50)
Unknown	17,400 (NA)	6598 (NA)	2111 (NA)	815 (NA)
Smoking^a^				
Habitual smoking	21,904 (26.53)	6503 (26.44)	2078 (25.93)	701 (26.06)
Non-habitual smoking	60,652 (73.47)	18,096 (73.56)	5936 (74.07)	1989 (73.94)
Unknown	19,726 (NA)	7267 (NA)	2322 (NA)	880 (NA)
Low-dose aspirin				
Presence	119 (0.12)	38 (0.12)	12 (0.12)	4 (0.11)
Absence	102,163 (99.88)	31,829 (99.88)	10,324 (99.88)	3566 (99.89)
Endoscopies				
Presence	10,030 (9.81)	5388 (16.91)	1839 (17.80)	813 (22.77)
Absence	92,252 (90.19)	26,478 (83.09)	8496 (82.20)	2757 (77.23)
Second-line eradication				
Presence	9275 (9.07)	2772 (8.70)	708 (6.85)	262 (7.33)
Absence	93,007 (90.93)	29,095 (91.30)	9628 (93.15)	3309 (92.67)
Index year				
2013	0 (0.0)	0 (0.0)	0 (0.0)	0 (0.0)
2014	6321 (6.18)	1982 (6.22)	620 (6.00)	222 (6.21)
2015	6990 (6.83)	2191 (6.88)	681 (6.59)	236 (6.62)
2016	9687 (9.47)	3025 (9.49)	978 (9.46)	323 (9.06)
2017	11,672 (11.41)	3625 (11.38)	1140 (11.03)	402 (11.26)
2018	14,372 (14.05)	4484 (14.07)	1479 (14.31)	502 (14.06)
2019	15,782 (15.43)	4898 (15.37)	1604 (15.52)	559 (15.65)
2020	13,421 (13.12)	4191 (13.15)	1345 (13.01)	448 (12.56)
2021	14,979 (14.64)	4657 (14.61)	1555 (15.04)	546 (15.29)
2022	9059 (8.86)	2813 (8.83)	934 (9.04)	332 (9.30)

Data are *N* (%), unless otherwise specified

*BMI* Body mass index, *DU* Duodenal ulcer, *GDU* Gastric ulcer and duodenal ulcer, *GU* Gastric ulcer, *HPAG* *Helicobacter pylori*–associated gastritis, *max* maximum, *min* minimum, *NA* not available; Q1/3, first/third quartile

^a^The denominator was the number of patients from total excluding unknown

### Risk of gastric cancer following *H. pylori* eradication therapy across different groups

In the crude cohort, statistically significant differences were observed in HRs between HPAG and DU (HR using the latter as a reference [95%CI]: 2.22 [1.48–3.35]; *p* < 0.001) and between GU and DU (2.89 [1.89–4.41]; *p* < 0.001) (Table 2), as well as between GU and HPAG (1.31 [1.11–1.54]; *p* = 0.001) and between GDU and GU (0.44 [0.24–0.80]; *p* = 0.007) (Table A2). In the weighted cohort, statistically significant differences were observed in HRs between HPAG and DU (HR using the latter as a reference [95%CI]: 2.03 [1.31–3.13]; *p* = 0.001) and between GU and DU (2.37 [1.52–3.71]; *p* < 0.001) (Table 2), as well as between GDU and GU (0.48 [0.24–0.93]; *p* = 0.029) (Table A2). Of the combinations with statistically significant differences in the crude cohort, a statistically significant difference was not observed between GU and HPAG in the weighted cohort.

**Table 2:** Hazard ratio of gastric cancer by each group vs. DU

	**Reference**	**HR (95%CI)**	***p-*value**
**Crude cohort**			
HPAG	(vs. DU)	2.22 (1.48–3.35)	< 0.001
GU		2.89 (1.89–4.41)	< 0.001
DU		NA	NA
GDU		1.27 (0.62–2.58)	0.518
**Weighted cohort**			
HPAG	(vs. DU)	2.03 (1.31–3.13)	0.001
GU		2.37 (1.52–3.71)	< 0.001
DU		NA	NA
GDU		1.13 (0.52–2.46)	0.761

*CI* Confidence interval, *DU* Duodenal ulcer, *GDU* Gastric ulcer and duodenal ulcer; GU, gastric ulcer; HPAG, *Helicobacter pylori*–associated gastritis; HR, hazard ratio; NA, not applicable

A similar trend was shown in a sensitivity analysis using lag time set to 2 or 3 years (Table A3).

### Cumulative probability of development of gastric cancer at median follow-up period

**Fig. 3:**
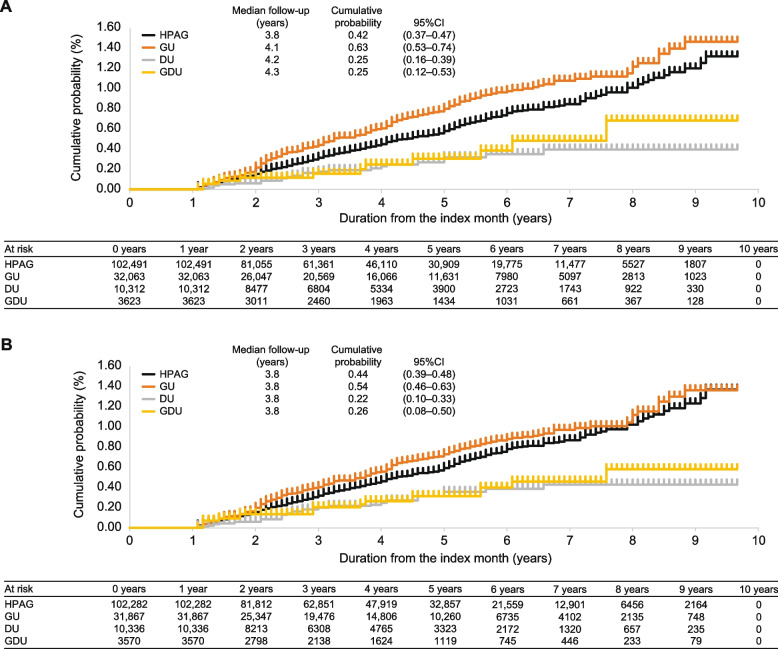
Cumulative probability of development of gastric cancer. (**A**) Crude cohort. (**B**) Weighted cohort. CI, confidence interval; DU, duodenal ulcer; GDU, gastric ulcer and duodenal ulcer; GU, gastric ulcer; HPAG, *Helicobacter pylori*–associated gastritis

The cumulative probability of development of gastric cancer over time is shown in Fig. 3. In the crude cohort, cumulative probabilities (95%CI) per the median follow-up years were 0.42% (0.37–0.47)/3.8 years for HPAG, 0.63% (0.53–0.74)/4.1 years for GU, 0.25% (0.16–0.39)/4.2 years for DU, and 0.25% (0.12–0.53)/4.3 years for GDU. In the weighted cohort, cumulative probabilities (95%CI) per the median follow-up years (3.8 years for all) were 0.44% (0.39–0.48), 0.54% (0.46–0.63), 0.22% (0.10–0.33), and 0.26% (0.08–0.50), respectively.

A similar trend was observed for all groups in the sensitivity analysis with the lag time set to 2 and 3 years (Fig. A1). The cumulative probabilities per the median follow-up years (4.5 years for all) with the lag time set to 2 years were 0.37% for HPAG, 0.47% for GU, 0.21% for DU, and 0.19% for GDU. The cumulative probabilities per the median follow-up years (5.2 years for all) with the lag time set to 3 years were 0.31%, 0.36%, 0.16%, and 0.11%, respectively.

## Discussion

This is the first nationwide, large-scale, retrospective cohort study to investigate gastric cancer risk after *H. pylori* eradication therapy among patients with HPAG, GU, DU, and GDU using the JMDC Claims Database, which is a database of health insurance claims that is cumulated from approximately 17 million beneficiaries in Japan. In the weighted cohort (after propensity matching), the HRs of gastric cancer and the cumulative probabilities of development of gastric cancer at median follow-up period demonstrated that the incidence of gastric cancer after *H. pylori* eradication therapy was higher in the HPAG and GU groups compared with the DU group.

In Japan, with the expansion of insurance coverage for *H. pylori* eradication therapy, the incidence of gastric cancer remains high [15]. Most patients infected with *H. pylori* have premalignant conditions, including gastric atrophy and intestinal metaplasia [5, 16]. Gastric atrophy and the degree of atrophy at the time of *H. pylori* eradication therapy are known risk factors for gastric cancer development even after *H. pylori* eradication therapy [10, 17–19]. The degree of gastric atrophy is greater in patients with chronic gastritis and GU compared with patients with DU [7, 8, 20, 21]. In a prospective study in Japan, Kamada et al. reported that the incidence of gastric cancer after *H. pylori* eradication therapy was 2.1% (12/575) for GU, 0.4% (2/453) for atrophic gastritis, and 0% (0/654) for DU [9]. Moreover, several studies have indicated that patients with advanced grades of atrophy and intestinal metaplasia had an increased risk of developing gastric cancer even after successful eradication of *H. pylori* [9, 22, 23]. In the current study, gastric cancer risk after *H. pylori* eradication therapy was higher in patients with GU and HPAG compared with patients with DU, consistent with results reported previously [9]. Our data also show that the risk of gastric cancer continues even after eradication (especially for patients with GU and HPAG), suggesting that regular surveillance, such as with endoscopic evaluation, should be recommended.

In patients with successful *H. pylori* eradication therapy, gastric cancer risk is decreased [24–27], but the risk remains higher than in people never infected with *H. pylori* [1, 23]. Late eradication of *H. pylori* is one risk factor for gastric cancer occurring after eradication therapy [22]. This suggests that the gastric cancer risk that has accumulated prior to eradication may remain to some extent after the eradication therapy. In this study, the lag time was defined as 1 year. Although the Japanese healthcare system is considered to have good diagnostic capabilities [24] and technical support for endoscopic examinations has been developing [28, 29], gastric cancer within 1 year after eradication is common [30]. Gastric cancers detected within 1 year after eradication therapy may have developed before the eradication therapy and been overlooked on previous endoscopy [29, 30]. Hansson et al. reported that the incidence of gastric cancer in patients with GU was higher than that of the Swedish general population during the first 3 years of follow-up and remained significantly increased throughout follow-up; however, the incidence of gastric cancer was significantly lower in patients with DU compared with the Swedish general population [31]. Furthermore, Japanese research reported that gastric cancer was commonly detected within 3 years after successful *H. pylori* eradication [30, 32]. In this study, to evaluate the impact of missed gastric cancers on the cumulative probability of development of gastric cancer, sensitivity analyses were conducted with a lag time of 2 or 3 years. The cumulative probabilities per the median follow-up years with a 1-year lag time were similar to those with a lag time of 2 and 3 years, suggesting that the results would be less affected by missed gastric cancers in this study. Furthermore, the incidence of metachronous gastric cancer after endoscopic submucosal dissection (ESD) was similar for patients who had *H. pylori* eradication > 1 year before ESD and those who had eradication therapy after ESD for 4.1 years as the median follow-up period [33]. However, it should be noted that in a randomized trial, the incidence of metachronous gastric cancer detected on endoscopy performed > 1 year after treatment (median follow-up period 5.9 years) was lower for patients who received *H. pylori* eradication therapy with antibiotics compared with placebo [34]. Nevertheless, because it is difficult to eliminate gastric cancer risk and the risk remains after successful *H. pylori* eradication therapy, regular surveillance, primarily by endoscopy, is strongly recommended [1]. Frequent endoscopy is recommended during the first year after successful *H. pylori* eradication therapy to improve the accuracy of detection of gastric cancer [35]. Furthermore, consideration that the gastric cancer may have been overlooked before the eradication therapy is important, especially immediately after *H. pylori* eradication therapy. In this study, the most common frequency of endoscopy was once per year in all groups (> 90%), and there was no difference in the frequency of endoscopy between the groups, suggesting that our results are not due to differences in endoscopy frequency immediately after *H. pylori* eradication therapy.

This study aimed to determine the difference in gastric cancer development among patients with HPAG, GU, DU, and GDU after *H. pylori* eradication. Gastric cancer due to *H. pylori* is associated with inflammation caused by the infection and subsequent mucosal changes. The degree of mucosal atrophy and intestinal metaplasia after inflammation are related to the level of acid secretion and are considered risk factors for gastric cancer development. Patients with GU have generally low gastric acidity, while patients with DU have high gastric acidity [36]. In patients with GDU, GU is often located near the pyloric gland [37, 38], and it has been shown that the closer the ulcer site is to the pylorus, the higher the level of acid secretion [39]. These reports have suggested that acid secretion ability is maintained in GDU. Moreover, in a previous report that evaluated the degree of atrophy by ulcer based on the pepsinogen I/II ratio, an indicator of gastric mucosal atrophy, and the actual endoscopic atrophy classification (Kimura-Takemoto classification), the degree of atrophy in patients with GDU was lower than that in patients with GU and closer to that in patients with DU [20]. Earlier studies also support the lower degree of atrophy in patients with GDU [40, 41]. Our study was a retrospective database study and we were not able to evaluate mucosal atrophy; however, based on the above evidence, ulceration can be taken as an indicator of degree of mucosal atrophy, and our data, which showed that the risk of gastric cancer was lower in GDU than in GU, are probably due to the differences in the degree of mucosal atrophy.

Several limitations of the study need to be considered. As this was a non-randomized study, selection bias or confounding cannot be excluded. For minimizing the effect of confounding on the results, a propensity score approach was used for the analyses. However, because the study was retrospective, residual confounding may exist—for example, factors such as the presence or severity of gastric atrophy, history of gastric cancer, adherence to *H. pylori* eradication therapy, and outcome of eradication therapy could not be fully accounted for. Because the database did not include the results of *H. pylori* infection diagnosis, patients may have been misclassified. However, misclassification was minimized by including only patients who had undergone *H. pylori* testing before first-line eradication. Moreover, in Japan, most patients who fail first-line eradication therapy receive second-line eradication therapy, and the success rates for first-line and second-line eradication therapies are approximately 90% for each [42, 43]. In the study, 7.27–9.03% of patients across the groups received second-line eradication, suggesting that *H. pylori* eradication was successful in almost all of the analysis populations. Patients who were diagnosed with cancer, including gastric cancer, 1 year before or after the index month were excluded from the study (0.43%, 0.75%, 0.11%, and 0.18% for HPAG, GU, DU, and GDU, respectively), but patients with an earlier history of gastric cancer were not excluded, and this may have impacted the cancer risk observed in this study. However, we believe that if the number of patients who underwent *H. pylori* eradication for the treatment of gastric cancer was very small (< 0.5%), as shown in previous studies of Japanese real-world data [44, 45], this is unlikely to have impacted the cancer risk observed in this study. Although the median follow-up period of the study was 3.8 years, gastric cancer may take longer to develop post eradication, and a longer follow-up period would provide an understanding of the long-term risks. In the JMDC Claims Database, data were collected for reimbursement purposes, and therefore important information—including disease severity, family history, reason for prescription, or adherence—may be missing. Furthermore, because diagnosis was classified by the ICD-10 codes in the database and clinical observations were not confirmed, misclassification of conditions like gastric atrophy or *H. pylori* infection could impact the results. In addition, although the JMDC database enabled us to track patients across different medical institutions, if patients changed health insurance associations, the medical records were not carried over. For investigating differences in the gastric cancer risk, the follow-up period was relatively short. Additionally, the results may not be generalizable to the population in Japan because the JMDC Claims Database includes mainly employees and their families, and data for individuals after the mandatory retirement age of 65 are limited.

## Conclusions

Patients with HPAG and GU were at a higher risk of gastric cancer compared with patients with DU, indicating that gastric atrophy remains a residual risk factor after *H. pylori* eradication therapy. Careful monitoring, such as by endoscopic examination, is required after successful eradication of *H. pylori* in patients at higher risk.

## Supplementary Information

Additional file 1

## Data Availability

The data analyzed in this study were obtained from JMDC Inc. (https://www.jmdc.co.jp/en/). The data are available from JMDC Inc. but were used under license for the current study; therefore, restrictions apply, and the data are not publicly available. For inquiries about access to the data set used in this study, please contact JMDC (https://www.jmdc.co.jp//en/inquiry/).

## Abbreviations

GU: Gastric ulcer
DU: Duodenal ulcer
MALT: Mucosa–associated lymphoid tissue
HPAG: *Helicobacter pylori*–associated gastritis
GDU: Gastric ulcer and duodenal ulcer
ICD-10: International Classification of Diseases 10th Revision
BMI: Body mass index
CI: Confidence interval
HR: Hazard ratio
ESD: Endoscopic submucosal dissection
