# Supplementary material for: Gastric cancer risk after Helicobacter pylori eradication in gastritis and peptic ulcer: a retrospective cohort study in Japan
Source: BMC Gastroenterol. 2025 Jul 1;25:463. doi: 10.1186/s12876-025-04034-3 (PMC12211893; doi:10.1186/s12876-025-04034-3)

Supplementary Information

TITLE

Gastric cancer risk after *Helicobacter pylori* eradication in gastritis and peptic ulcer

AUTHORS

Kentaro Sugano^1^, Chihiro Suzuki^2^, Mihoko Ota^2†^, Ryuichi Iwakiri^3^

AUTHORS’ AFFILIATIONS

^1^Jichi Medical University, Tochigi, Japan

^2^Japan Medical Office, Takeda Pharmaceutical Company Limited, Tokyo, Japan

^3^Department of Gastroenterology, Shin Komonji Hospital, Fukuoka, Japan

^†^Current affiliation: Institute of Science Tokyo, Tokyo, Japan

**Table A1** Standardized differences in baseline characteristics between disease groups

**Table A2** Hazard ratio of gastric cancer by each group vs. HPAG, GU, and GDU

**Table A3** Hazard ratio of gastric cancer by each group with 2-year or 3-year lag-time period

**Fig. A1** Cumulative probability of development of gastric cancer in weighted cohort

**Table A1** Standardized differences in baseline characteristics between disease groups

|  | **HPAG vs. GU** | **HPAG vs. DU** | **HPAG vs. GDU** | **GU vs. DU** | **GU vs. GDU** | **DU vs. GDU** | |
| --- | --- | --- | --- | --- | --- | --- | --- |
| **Crude cohort** |  |  |  |  |  |  | |
| Sex | 0.10 | 0.31 | 0.40 | 0.22 | 0.30 | 0.09 | |
| Age (years), continuous | 0.08 | 0.27 | 0.14 | 0.34 | 0.22 | 0.12 | |
| Age (years), categorical | 0.08 | 0.22 | 0.09 | 0.30 | 0.17 | 0.13 | |
| BMI (kg/m^2^) | 0.05 | 0.04 | 0.07 | 0.09 | 0.08 | 0.09 | |
| Smoking | 0.29 | 0.37 | 0.49 | 0.08 | 0.19 | 0.11 | |
| Low-dose aspirin | 0.03 | 0.00 | 0.02 | 0.03 | 0.01 | 0.03 | |
| Index year | 0.21 | 0.18 | 0.26 | 0.04 | 0.07 | 0.09 | |
| **Weighted cohort** | |  | |  | | |  |
| Sex | 0.00 | 0.02 | 0.01 | 0.02 | 0.01 | 0.02 | |
| Age (years), continuous | 0.01 | 0.05 | 0.06 | 0.05 | 0.06 | 0.01 | |
| Age (years), categorical | 0.02 | 0.05 | 0.09 | 0.04 | 0.07 | 0.04 | |
| BMI (kg/m^2^) | 0.05 | 0.02 | 0.04 | 0.07 | 0.03 | 0.05 | |
| Smoking | 0.00 | 0.01 | 0.01 | 0.01 | 0.01 | 0.00 | |
| Low-dose aspirin | 0.00 | 0.00 | 0.00 | 0.00 | 0.00 | 0.00 | |
| Index year | 0.00 | 0.02 | 0.03 | 0.02 | 0.03 | 0.02 | |

BMI, body mass index; DU, duodenal ulcer; GDU, gastric ulcer and duodenal ulcer; GU, gastric ulcer; HPAG, *Helicobacter pylori*–associated gastritis

**Table A2** Hazard ratio of gastric cancer by each group vs. HPAG, GU, and GDU

|  | **Reference** | **HR (95%CI)** | ***p-*value** |
| --- | --- | --- | --- |
| **Crude cohort** | | | |
| HPAG | (vs. HPAG) | NA | NA |
| GU |  | 1.31 (1.11–1.54) | 0.001 |
| DU |  | 0.45 (0.30–0.68) | <0.001 |
| GDU |  | 0.56 (0.31–1.03) | 0.061 |
| HPAG | (vs. GU) | 0.77 (0.65–0.90) | 0.001 |
| GU |  | NA | NA |
| DU |  | 0.35 (0.23–0.53) | <0.001 |
| GDU |  | 0.44 (0.24–0.80) | 0.007 |
| HPAG | (vs. GDU) | 1.77 (0.97–3.22) | 0.061 |
| GU |  | 2.29 (1.25–4.19) | 0.007 |
| DU |  | 0.79 (0.39–1.61) | 0.518 |
| GDU |  | NA | NA |
| **Weighted cohort** | | | |
| HPAG | (vs. HPAG) | NA | NA |
| GU |  | 1.17 (0.99–1.38) | 0.067 |
| DU |  | 0.49 (0.32–0.76) | 0.001 |
| GDU |  | 0.56 (0.29–1.08) | 0.081 |
| HPAG | (vs. GU) | 0.86 (0.72–1.01) | 0.067 |
| GU |  | NA | NA |
| DU |  | 0.42 (0.27–0.66) | <0.001 |
| GDU |  | 0.48 (0.24–0.93) | 0.029 |
| HPAG | (vs. GDU) | 1.80 (0.93–3.48) | 0.081 |
| GU |  | 2.10 (1.08–4.10) | 0.029 |
| DU |  | 0.89 (0.41–1.93) | 0.761 |
| GDU |  | NA | NA |

CI, confidence interval; DU, duodenal ulcer; GDU, gastric ulcer and duodenal ulcer; GU, gastric ulcer; HPAG, *Helicobacter pylori*–associated gastritis; HR, hazard ratio; NA, not applicable

**Table A3** Hazard ratio of gastric cancer by each group with 2-year or 3-year lag-time period

(A) 2-year lag-time period

|  | **Reference** | **HR (95%CI)** | ***p*-value** |
| --- | --- | --- | --- |
| **Crude cohort** | | | |
| HPAG | (vs. HPAG) | NA | NA |
| GU |  | 1.27 (1.05–1.54) | 0.016 |
| DU |  | 0.46 (0.29–0.75) | 0.002 |
| GDU |  | 0.49 (0.23–1.03) | 0.060 |
| HPAG | (vs. GU) | 0.79 (0.65–0.96) | 0.016 |
| GU |  | NA | NA |
| DU |  | 0.37 (0.23–0.60) | <0.001 |
| GDU |  | 0.39 (0.18–0.83) | 0.015 |
| HPAG | (vs. DU) | 2.15 (1.34–3.46) | 0.002 |
| GU |  | 2.72 (1.67–4.44) | <0.001 |
| DU |  | NA | NA |
| GDU |  | 1.06 (0.44–2.54) | 0.896 |
| HPAG | (vs. GDU) | 2.05 (0.97–4.33) | 0.060 |
| GU |  | 2.57 (1.21–5.49) | 0.015 |
| DU |  | 0.94 (0.39–2.26) | 0.896 |
| GDU |  | NA | NA |
| **Weighted cohort** | | | |
| HPAG | (vs. HPAG) | NA | NA |
| GU |  | 1.14 (0.93–1.39) | 0.203 |
| DU |  | 0.54 (0.33–0.90) | 0.017 |
| GDU |  | 0.43 (0.18–1.00) | 0.050 |
| HPAG | (vs. GU) | 0.88 (0.72–1.07) | 0.203 |
| GU |  | NA | NA |
| DU |  | 0.48 (0.28–0.80) | 0.005 |
| GDU |  | 0.37 (0.16–0.89) | 0.026 |
| HPAG | (vs. DU) | 1.85 (1.12–3.06) | 0.017 |
| GU |  | 2.10 (1.25–3.53) | 0.005 |
| DU |  | NA | NA |
| GDU |  | 0.79 (0.29–2.09) | 0.629 |
| HPAG | (vs. GDU) | 2.35 (1.00–5.53) | 0.050 |
| GU |  | 2.68 (1.13–6.35) | 0.026 |
| DU |  | 1.27 (0.48–3.39) | 0.629 |
| GDU |  | NA | NA |

CI, confidence interval; DU, duodenal ulcer; GDU, gastric ulcer and duodenal ulcer; GU, gastric ulcer; HPAG, *Helicobacter pylori*–associated gastritis; HR, hazard ratio; NA, not applicable

(B) 3-year lag-time period

|  | **Reference** | **HR (95%CI)** | ***p*-value** |
| --- | --- | --- | --- |
| **Crude cohort** | | | |
| HPAG | (vs. HPAG) | NA | NA |
| GU |  | 1.19 (0.93–1.52) | 0.159 |
| DU |  | 0.34 (0.17–0.66) | 0.002 |
| GDU |  | 0.61 (0.27–1.37) | 0.228 |
| HPAG | (vs. GU) | 0.84 (0.66–1.07) | 0.159 |
| GU |  | NA | NA |
| DU |  | 0.29 (0.15–0.57) | <0.001 |
| GDU |  | 0.51 (0.23–1.17) | 0.114 |
| HPAG | (vs. DU) | 2.94 (1.51–5.72) | 0.002 |
| GU |  | 3.48 (1.76–6.89) | <0.001 |
| DU |  | NA | NA |
| GDU |  | 1.79 (0.64–5.04) | 0.268 |
| HPAG | (vs. GDU) | 1.65 (0.73–3.71) | 0.228 |
| GU |  | 1.95 (0.85–4.44) | 0.114 |
| DU |  | 0.56 (0.20–1.57) | 0.268 |
| GDU |  | NA | NA |
| **Weighted cohort** | | | |
| HPAG | (vs. HPAG) | NA | NA |
| GU |  | 1.06 (0.83–1.36) | 0.641 |
| DU |  | 0.37 (0.18–0.76) | 0.007 |
| GDU |  | 0.44 (0.19–1.00) | 0.051 |
| HPAG | (vs. GU) | 0.94 (0.73–1.21) | 0.641 |
| GU |  | NA | NA |
| DU |  | 0.35 (0.17–0.73) | 0.005 |
| GDU |  | 0.41 (0.18–0.96) | 0.039 |
| HPAG | (vs. DU) | 2.70 (1.32–5.53) | 0.007 |
| GU |  | 2.87 (1.38–5.96) | 0.005 |
| DU |  | NA | NA |
| GDU |  | 1.17 (0.40–3.47) | 0.771 |
| HPAG | (vs. GDU) | 2.30 (1.00–5.30) | 0.051 |
| GU |  | 2.44 (1.04–5.71) | 0.039 |
| DU |  | 0.85 (0.29–2.51) | 0.771 |
| GDU |  | NA | NA |

CI, confidence interval; DU, duodenal ulcer; GDU, gastric ulcer and duodenal ulcer; GU, gastric ulcer; HPAG, *Helicobacter pylori*–associated gastritis; HR, hazard ratio; NA, not applicable

**Fig. A1** Cumulative probability of development of gastric cancer in weighted cohort.
(**A**) 2-year lag-time period. (**B**) 3-year lag-time period. CI, confidence interval; DU, duodenal ulcer; GDU, gastric ulcer and duodenal ulcer; GU, gastric ulcer; HPAG, *Helicobacter pylori*–associated gastritis


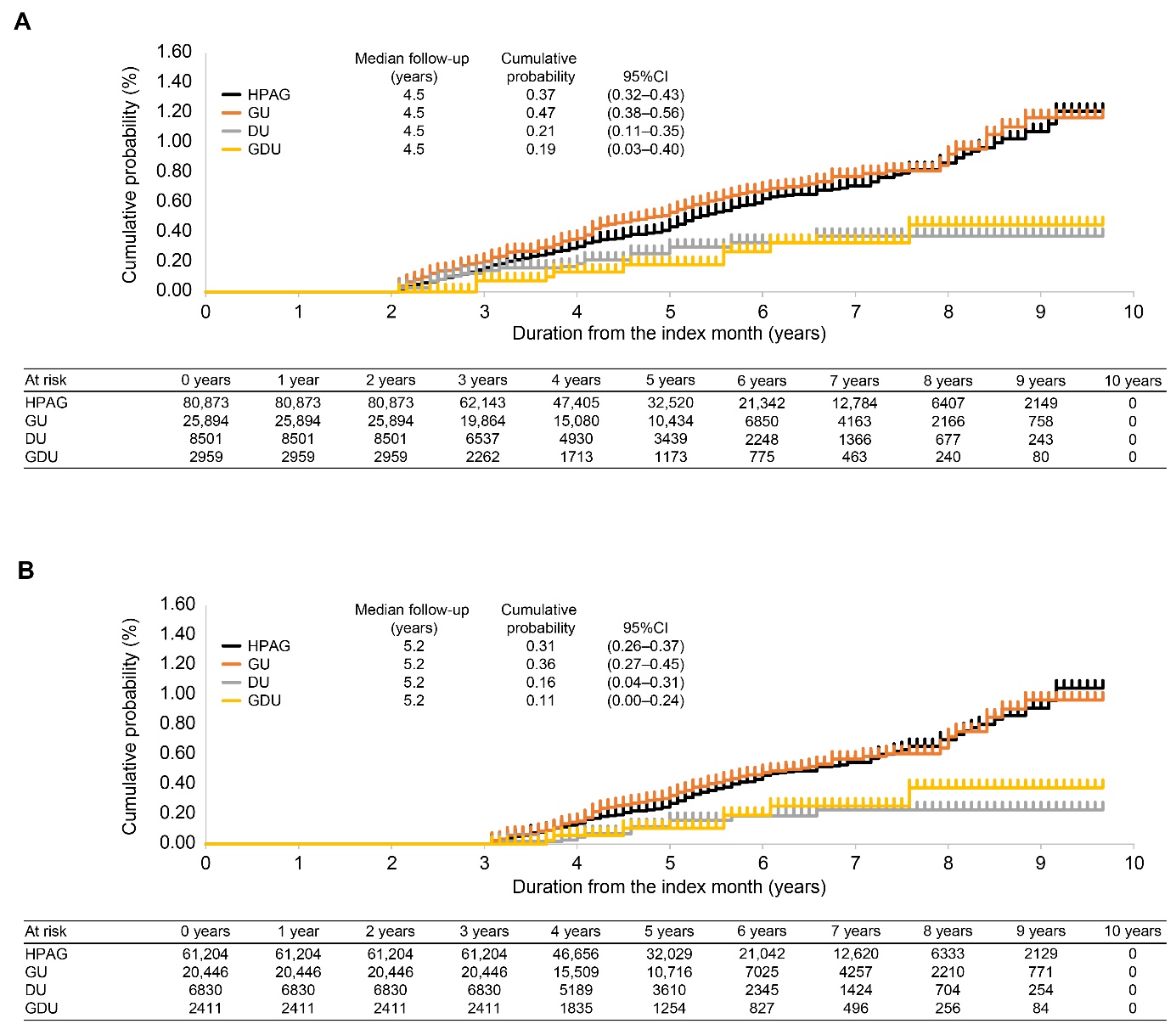

Supplement: Supplementary file 1 — Additional file 1 [file 12876_2025_4034_MOESM1_ESM.docx]
